# Foreign Body-induced Appendicitis in Situs Inversus Totalis: Diagnostic and Management Challenges in the Emergency Department: a Rare Case Report

**DOI:** 10.1097/MS9.0000000000003369

**Published:** 2025-05-20

**Authors:** Rahul Yadav, Sanish Pokhrel, Raman Kumar Gurmaita, Bhawana Aryal, Madan Thapaliya

**Affiliations:** a Universal College of Medical Sciences and Teaching Hospital, Ranigaon, Bhairahawa, Nepal; b Nepal Medical College and Teaching Hospital, Atterkhel, Kathmandu, Nepal; c Rapti Academy of Health Sciences, Dang, Nepal

**Keywords:** case report, dextrocardia, foreign body appendicitis, situs inversus totalis

## Abstract

**Introduction:**

Situs inversus totalis (SIT) is a rare congenital condition in which the major visceral organs are mirrored from their normal positions. Appendicitis in patients with SIT can be diagnostically challenging due to the atypical location of abdominal pain, typically presenting on the left side. Foreign body impacted appendicitis is a rare occurrence.

**Case presentation:**

We present a case of a 10-year-old boy presented to the emergency room with complaints of abdominal pain in the left lower abdomen. Acute appendicitis with an impacted foreign body in the lumen of the appendix was found in ultrasonography (USG). Chest X-ray showed dextrocardia and fundic gas under the right hemidiaphragm and the liver shadow on the left illustrating SIT. The patient underwent an urgent midline laparotomy, revealing a left iliac fossa appendix consistent with situs inversus. The inflamed appendix contained a palpable foreign body. The appendix was excised, revealing a sewing machine pin approximately 5 cm in length lodged in the appendiceal lumen.

**Discussion:**

Situs inversus totalis is a rare condition in which the orientation of visceral organs mirrors the normal anatomy. Abdominal pain is one of the most frequent chief complaints among patients in the Emergency department, with appendicitis being the most common surgical condition diagnosed. Left-sided lower abdominal pain can be the presentation of appendicitis in situs inversus totalis. Usual presentation is right-sided pain so doctors do not consider left-sided lower abdominal pain as differential diagnosis of acute appendicitis making atypical presentation difficult to diagnose. Failure to consider this anatomical variation could delay diagnosis, leading to misinterpretation of symptoms and unnecessary or inappropriate investigations.

**Conclusion:**

In cases of left lower quadrant pain with dextrocardia and right-sided gastric gas bubble on chest X-ray should be thought of acute appendicitis. USG or computed tomography abdomen must be done to diagnose appendicitis with an impacted foreign body in SIT.

**HIGHLIGHTS**

- Appendicitis in patients with situs inversus totalis can be diagnostically challenging due to the atypical location of abdominal pain, typically presenting on the left side.
- Foreign-body-induced appendicitis is a rare occurrence in the Emergency department.
- The aim of treatment is to minimize the risk of complications such as perforation, making appendectomy necessary.

## INTRODUCTION

Situs inversus totalis (SIT) is a rare congenital condition in which the major visceral organs are mirrored from their normal positions, occurring in approximately 1 in 10,000 individuals, and is more common in males, with a male-to-female ratio of 1.5:1^[1]^. The exact cause is unknown, but both autosomal recessive and X-linked inheritance patterns have been observed. It may also occur in association with syndromes like Kartagener syndrome or Primary Ciliary Dyskinesia^[2]^. Appendicitis in patients with situs inversus can be diagnostically challenging due to the atypical location of abdominal pain, typically presenting on the left side^[3]^. Foreign body ingestion is common in children. Ingestion of foreign bodies is common in pediatric patients, especially those under 4 years old, with 98% of cases being accidental. Most foreign bodies, typically found at home, pass without complications, being expelled in feces within a few days. However, 10–20% may require endoscopic removal, and less than 1% may need surgery^[4]^. The occurrence of a foreign body migrating to and causing appendicitis is rarest, particularly in pediatric patients.

Here, we present an unusual case of appendicitis in a child with SIT, complicated by the presence of a sewing machine pin in the appendix. This case has been reported in line with the 2023 Surgical Case Report (SCARE) guidelines^[5]^.

## CASE REPORT

A 10-year-old boy from a rural area in Nepal presented to the Emergency department (ED) of a tertiary care centre with the complaint of pain in the left iliac fossa for 2 days. Pain was acute in onset, sharp, non-radiating, aggravated on movements and relieved on rest. A single episode of vomiting was present which contained food particles, non projectile, non bile or blood stained which was followed by fever with maximum recorded temperature of 101°F. On examination, his BP was 110/80 mmHg, PR 90 bpm, RR 20/min, Temperature 98.9°F, and SpO_2_ 99%. Tenderness was present in the left iliac fossa, and rebound tenderness was present, with guarding also present. Laboratory investigations showed that he had WBC count of 13,800/mm^3^, Neutrophils 80%, Lymphocytes 12%, Eosinophil 2%, monocytes 3%, Hemoglobin 10.7 gm %, Platelets 225 000 mm^3^, PT 14 s, and INR 1.08.

**Figure 1.**
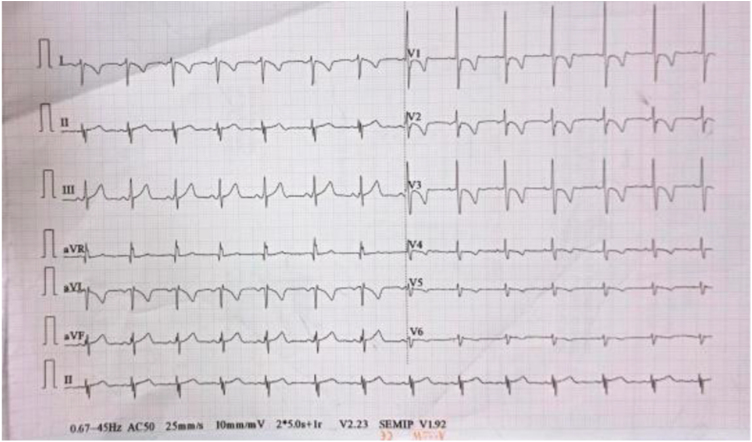
ECG showing features of dextrocardia.

The standard 12-lead electrocardiogram was done in ER which revealed unusual features: Right axis deviation, P-wave, QRS complex, and T-wave in lead I were inverted. There is poor progression of the R wave across the precordial leads, with the tallest R waves appearing in V1 and V3, while leads V5 and V6 show small R waves (Fig. 1). Diminished voltage in leads V5 and V6, which usually display the tallest R waves in a normal ECG, show diminished voltage. There are no ST segment elevations or depressions that would suggest acute ischemia. An ECG is a part of standard workup for acute abdominal pain to rule out any potential cardiac involvement or abnormalities, given that some cardiac conditions can present with referred pain to the abdomen.

In this case, confirming an ECG suggesting dextrocardia with no other abnormalities helped us to narrow the differential diagnosis and focus on other causes of patient symptoms, ultimately leading to the diagnosis of appendicitis.

**Figure 2.**
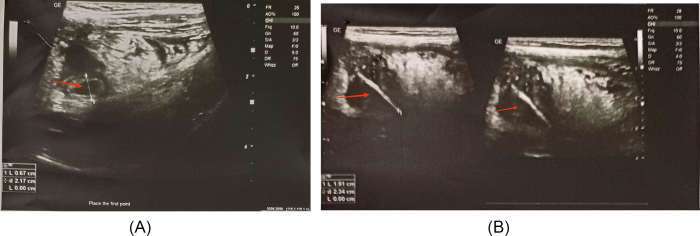
(A) tubular structure with thickened hypervascular walls with lumen measures 6-7 mm in caliber. Hyperechoic omental cap is also noted surrounding the tubular lumen. (B) Appendicular lump with foreign body in the lumen of appendix.

Patient was sent for ultrasonography (USG) of abdomen and pelvis which showed probe tenderness in the left lower quadrant with findings of an appendicular lump with an echogenic structure about 3.5 cm casting posterior acoustic shadow in the lumen of the appendix, suspected to be foreign body (Fig. 2).

**Figure 3.**
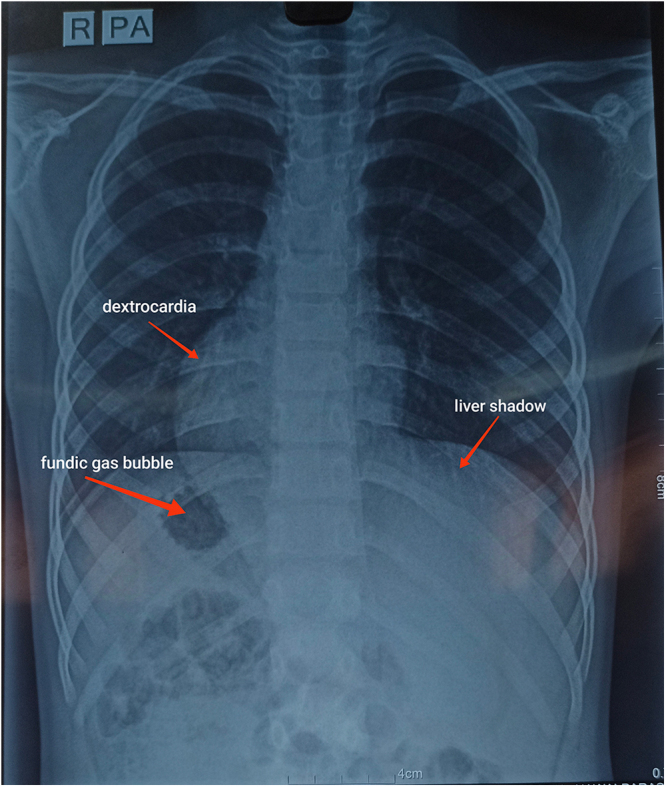
Chest X-ray PA view showing dextrocardia with aortic arch curving toward right, stomach fundic gas bubble under the right hemidiaphragm, liver shadow under left hemidiaphragm.

Chest X ray was done which shows cardiac shadow to the right (dextrocardia) with aortic arch curving toward right and stomach fundic gas bubble under the right hemidiaphragm suggesting SIT (Fig. 3).

The provisional diagnosis was foreign body-induced appendicitis in SIT.

At the ED, primary management was done. Patient was put on nil per oral, intravenous cannula was opened and IV fluids were given. Injection ketorolac 15 mg was given intravenously for pain relief, antibiotics, ceftriaxone 500 mg and ornidazole 250 mg was given intravenously. All preoperative preparation was done and the patient was sent for emergency surgery.

**Figure 4.**
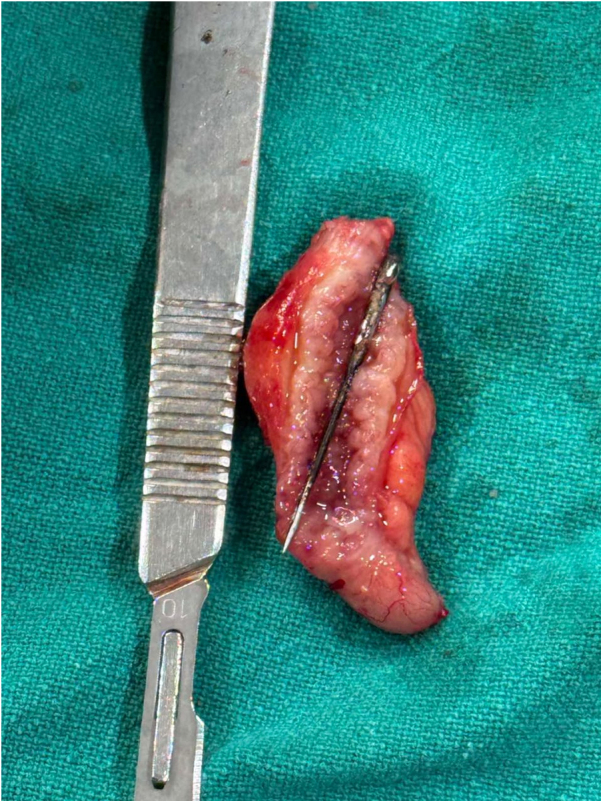
Sewing machine pin about 5 cm in the lumen of appendix.

The patient underwent urgent midline laparotomy because it allows direct visualization and removal of the foreign body, drainage of any infection, and assessment of surrounding structures. Intraoperatively, the appendix was located in the left iliac fossa, consistent with situs inversus. Upon dissection, the appendix appeared inflamed, and a foreign body was palpable within the lumen. The appendix was excised, revealing a sewing machine pin approximately 5 cm in length lodged in the appendiceal lumen (Fig. 4)

The patient remained stable during the postoperative period with no signs of complications. Pain was effectively managed with paracetamol and occasional doses of tramadol. Early mobilization was encouraged to prevent complications, and bowel movements resumed within 48 hours, indicating normal gastrointestinal recovery. The surgical site was clean with no signs of infection, erythema, discharge, or dehiscence.

The patient was advised to keep the wound clean and dry, with dressing changes every other day or when soiled. They were instructed to complete the prescribed antibiotics and take analgesics as needed. Strenuous activities and heavy lifting should be avoided for 4–6 weeks, with gradual increases in activity as tolerated. A regular diet can be resumed, starting with soft, easily digestible foods while maintaining proper hydration. The patient was advised to report any red flag signs, such as fever, severe pain, or excessive wound discharge, to the respective department immediately.

Follow-up was scheduled for 1 week to inspect the wound and remove sutures if necessary, with additional visits planned at 2 weeks and 1 month to monitor recovery and check for late complications like adhesions or bowel obstruction.

## DISCUSSION

SIT is an autosomal recessive congenital anomaly which occurs by a 270-degree clockwise rotation of the abdominal organs during embryonic development rather than counter clockwise, leading to their complete transposition across the abdominal cavity^[3]^.

Abdominal pain is a common emergency complaint, with appendicitis being the most frequent surgical diagnosis. Appendicitis is typically diagnosed based on clinical symptoms, physical examination, and WBC count. The MANTRELS score, which includes factors like pain migration, nausea, right lower quadrant tenderness, fever, and leukocytosis, aids in diagnosis. However, classic symptoms can be unreliable, and up to 33% of cases present atypically. Unusual left lower quadrant pain in appendicitis can lead to misdiagnosis or false-negative results^[6]^. Left lower quadrant pain in adolescent males has a wide differential diagnosis, including gastrointestinal issues (e.g., diverticulitis, obstruction, hernia, regional enteritis, hypothyroidism-related ileus). vascular causes (e.g., aneurysm, mesenteric ischemia), and genitourinary conditions (e.g., renal colic, cystitis, epididymitis, prostatitis, or testicular torsion). Additionally, left-sided appendicitis or atypical right-sided appendicitis should be considered^[7]^.

In the usual presentation, about 4–8% of all patients visiting the ED suffer from acute appendicitis. Usual presentation is right-sided pain so doctors usually do not consider left-sided lower abdominal pain as differential diagnosis of acute appendicitis making the atypical presentation difficult to diagnose^[8]^. Appendicitis is primarily caused by a blockage of the appendix’s lumen, often due to a fecalith. However, in rare cases, it can result from an ingested foreign body, as illustrated in our case. Previous study indicate that a wide range of objects have been found lodged in the appendix, including birdshot, screws, drill bits, needles, bone fragments, seeds, and toothpicks^[9]^.

Typically, ingested foreign bodies pass through the gastrointestinal tract without complications, making their presence in the appendix uncommon. It’s important to note that while foreign-body ingestion is a frequent issue among children, foreign-body-induced appendicitis occurs in only 0.005% of cases^[10]^. Plain radiographs are generally not effective for diagnosing appendicitis. However, detecting dextrocardia on a chest X-ray and observing a right-sided gastric bubble on an abdominal X-ray can be significant indicators for diagnosing SIT^[11]^.

The aim of treatment is to minimize the risk of complications such as perforation, which is particularly high with sharp and elongated objects, making appendectomy necessary^[9]^. Complicated appendicitis cases are often influenced by the size and shape of the foreign bodies. Blunt objects typically lead to appendicitis through inflammation or obstruction of the appendiceal lumen. On the other hand, elongated and sharp foreign bodies, which make up 75% of cases, are more prone to causing perforation, periappendiceal abscesses, and peritonitis^[12]^.

## CONCLUSION

A patient presented with Left lower quadrant abdominal pain along with dextrocardia and right sided gastric gas bubble on chest X-ray should be considered for acute appendicitis. Atypical pain, particularly when it deviates from usual clinical patterns, should prompt health care providers to consider possibilities beyond the typical anatomical framework. Early investigations i.e. chest X-ray, computed tomography (CT), USG or magnetic resonance imaging helps in recognition of conditions such as SIT, preventing delays in care, and ultimately improving patient outcomes. USG or CT abdomen should be performed to diagnose appendicitis with an impacted foreign body as well as SIT. Urgent appendectomy is necessary to prevent the risk of perforations or any other complications.

## Data Availability

The datasets generated during the study are publicly available, available upon reasonable request.

## Research Registration Unique Identifying Number (UIN)

This is NOT applied in our case report.
